# Community Resilience and COVID-19: A Fuzzy-Set Qualitative Comparative Analysis of Resilience Attributes in 16 Countries

**DOI:** 10.3390/ijerph20010474

**Published:** 2022-12-28

**Authors:** Fangxin Yi, Jun Jie Woo, Qiang Zhang

**Affiliations:** 1 Innovation Centre for Risk Governance/School of Social Development and Public Policy, Beijing Normal University, Beijing 100875, China; 2 Lee Kuan Yew School of Public Policy, National University of Singapore, Singapore 119007, Singapore

**Keywords:** public service, resilience framework, comparative study, fsQCA, COVID-19

## Abstract

The COVID-19 pandemic has caused massive disruptions to governments and societies across the world. While public healthcare systems have come under immense pressure, public trust in governments and institutions are also in decline. In this paper, we seek to assess the resilience of policy systems and processes in 16 countries during the COVID-19 pandemic through the use of fuzzy-set Qualitative Comparative Analysis (fsQCA). We focus specifically on robustness, preparedness, social capital, and institutional strength as key attributes of community resilience at city-level. Our analysis of the data reveals that COVID-19 resilience is dependent on a combination of factors, with a multi-factorial approach to policy design and governance necessary for effective pandemic and disaster recovery.

## 1. Introduction

The COVID-19 pandemic has given rise to severe disruptions and policy challenges for governments across the world. While public healthcare systems have come under immense pressure, public trust in governments and institutions are also in decline. As a consequence, there has been intensified focus on resilience as a policy goal or attribute in societies seeking to address the COVID-19 pandemic [1,2,3,4]. Yet, despite this proliferation of research interest in COVID-19 policy responses, much of this emerging work policy has consisted of single-case or small-n studies. There has, as yet, been little effort at developing large-n comparative case studies of countries from multiple regions across the world. This paper therefore seeks to address this gap in the literature by assessing the disaster response efforts and community resilience of 16 countries (Cambodia, China, France, India, Japan, Korea Rep., Malawi, Myanmar, New Zealand, Pakistan, Russia Federation, Singapore, Tanzania, United Kingdom, United States, Zimbabwe) during the COVID-19 pandemic through the use of fuzzy-set Qualitative Comparative Analysis (fsQCA).

As widely discussed in the literature related to resilience, the concept of “resilience” is borrowed from the ecological and physical sciences into social studies and public policy addressing the uncertainties and complexity. Drawing from an extensive review of the existing literature, we focus specifically on robustness, preparedness, social capital, and institutional strength as key attributes of community resilience concerning the physical aspect, social aspect, and institutional aspects. Based on these attributes and our data analysis, we identify four distinct combinations of community resilience across the 16 countries. Empirical results show that COVID-19 community resilience does not depend on any single factor or attribute. It is instead a function of multiple variables, with different countries and cities exhibiting varying extents of resilience based on different combinations of factors or attributes. In identifying these factors, we hope to shine light on the possible attributes that are necessary for resilient institutions and policy designs during a global pandemic. This will hopefully help stimulate further research into policy design for resilience.

In the following sections, we will first provide a review of the existing literature on resilience and policy design. We will also discuss some of the emerging research on community resilience and disaster recovery during the COVID-19 pandemic. This is followed by a description of our theoretical framework and methodology. We will then present our research findings and conclude with discussions and implications for future research.

## 2. Literature Review

### 2.1. Resilience and Policy Design

Resilience has long been an area of interest among policy scholars. This is particularly the case for studies in the ‘new design orientation’, with resilience seen as a goal that can be achieved through the incorporation of specific elements or aspects in policy designs [5,6,7]. Yet, due to its increasingly widespread use across a diverse array of policy domains, resilience as a policy concept is highly ‘malleable’ and lacking in definitional clarity [8].

Nonetheless, it is possible to trace out a faint outline of what resilience means to policy scholars, even if precise definitions of resilience attributes and/or outcomes tend to vary across policy domains, socio-geographical contexts, and even disciplinary persuasions.

Drawn from studies of socio-ecological systems, traditional understandings of resilience have focused on a system’s ability to ‘bounce back’ from a shock and return to a prior equilibrium state [9,10,11,12]. In fact, the etymology of the term resilience is the Latin word “resilio”, which means to “spring back”. At the heart of such understandings of resilience is an emphasis on stability, and consequently, the policy responses and measures necessary for maintaining this stability and bringing a system back to its original state in the event of a crisis or disruption [9,13,14,15].

This initial focus on resilience as rebounding has, however, been found to be problematic, with a return to pre-crisis conditions often resulting in the replication of the very institutional conditions or behavioral patterns that had given rise to the crisis in the first place [16]. This awareness of the limitations of resilience as rebounding has led policy scholars to seek out new approaches to conceptualizing and operationalizing resilience.

For instance, there has been growing emphasis on resilience as a matter of maintaining institutional and policy functionality, with resilient systems being those that are capable of maintaining institutional stability and public service delivery when faced with shocks, crises, and disruptions [17,18]. From this perspective, the emphasis is very much on withstanding external changes and shocks without any change in endogenous systemic function [19,20]. From a policy design perspective, this means replacing optimization with tolerance as guiding principle for policy design [21].

This focus on resilience as maintaining functionality frees policymakers and designers from having to return to pre-crisis conditions and focuses instead on tolerating the negative impacts of disruption and ultimately ‘living with’ crisis conditions by institutional adaptations or redesign [17,18]. This resonates strongly with emerging narrative of a ‘post-COVID’ reality that will be drastically different from pre-pandemic conditions [22,23,24].

It is, therefore, unsurprising that the COVID-19 pandemic would spark off a surge of scholarly interest in the differing extents of resilience in different societies across the world. In some instances, the focus has been on policy responses to COVID-19 and their impacts on economic and societal recovery [1,3,25,26]. At the heart of this is a concern with maintaining public service delivery and institutional integrity during the crisis [27]. Others have sought to understand the role of government resources or capacities in supporting COVID-19 policy responses and, hence, contributing to overall systemic resilience [25,28,29,30,31,32].

The concept of resilience has more recently found resonance among scholars of policy design, with a growing body of work that is focused on how policy design can contribute to resilience, as well as how the design process can give rise to resilient policy designs. The distinction between resilience as rebounding and resilience as functionality that has been discussed above features heavily in these studies, with some scholars defining resilience as a system’s ability to return to pre-crisis conditions while its ability to retain functionality during the crisis is referred to as robustness [17,18].

This distinction is given a temporal dimension by Howlett (2019) [33], who states that “individual policies can be thought of as both more or less robust—that is, capable of attaining their intended effects in a variety of circumstances—and more or less resilient—or capable of remaining robust over time”. In this instance, robustness refers to the ability to retain functionality during a crisis while resilience is associated with a system’s ability to maintain robustness, i.e., policy functionality, over extended periods of time.

This introduction of temporal dynamics addresses an important point in policy design that is often overlooked, i.e., the tendency for policy mixes to decline in effectiveness over time due to suboptimal processes of policy change such as layering, drift, and conversion that result in incoherence and/or inconsistency in the policy mix [34,35,36,37]. In any case, the point is made that there is a temporal aspect to resilience and policy design. This growing interest in resilience among scholars of policy design has a particularly strong bearing on notions of public service delivery.

### 2.2. Public Service Delivery and Slack

Contemporary approaches to public service delivery are very much dominated by what is known as the new public management (NPM) approach. NPM first emerged in the early 1990s, with early proponents of the movement focused on achieving greater efficiency and transparency in public administration through a reinvention of government that involved introducing private sector management practices into the public sector [38,39,40,41]. Emphasizing output measures of efficiency and effectiveness over traditional public administration’s focus on input and procedural considerations of equity and impartiality, NPM is very much centered on ensuring greater efficiency in the public sector, especially in resource optimization cost minimization [42]. Efforts to contract out the provision of certain public services or privatize public organizations are often carried out to reduce cost and ‘wastage’ in the public sector.

Scholars have identified three broad components of NPM: incentivizing, competition, and disaggregation [42,43]. These three components are common across existing understandings of NPM, although they can be further disaggregated into other constituent aspects. Studies on the application of NPM principles and practices to public healthcare have yielded mixed results. For instance, while the application of NPM to the UK’s National Healthcare System has allowed for more systematic measures of healthcare research output, it has also resulted in the “reduction of the rather intangible asset of medical knowledge creation into simple, recruitment-focused indicators”, with recruitment-based indicators often used as crude measures of medical knowledge creation [44].

Furthermore, despite NPM’s focus on efficiency and resource optimization, studies of public healthcare systems have found little evidence of efficiency gains from incorporating NPM practices into hospitals [45,46,47]. Another unintended consequence of introducing NPM into public healthcare systems, especially in terms of hospital corporatization and materialization, has been the emergence of rivalry and conflict among the various sets of stakeholders, particularly between medical professionals and managers [48,49,50].

While NPM has allowed governments to enhance their public service delivery mechanisms’ efficiency, it has also paradoxically compromised the resilience of these mechanisms and institutions. For instance, NPM’s emphasis on resource optimization had prompted many governments to take a ‘just-in-time’ approach to medical inventory management. This resulted in a shortage of PPEs and other crucial medical supplies during the COVID-19 pandemic in healthcare systems across the United States and Europe.

In contrast, countries that maintained a stockpile of medical supplies, such as Singapore and Finland, were able to ensure the availability of PPEs and, hence, enhance the resilience of their healthcare systems [51,52,53,54]. This has led to views that the resilience of a policy system may require building up excess capacity and organizational slack, which in turn involves setting aside funds and resources in anticipation of a potential crisis; this goes against the central tenet of NPM thinking that is resource optimization [30,31].

From a policy design perspective, it has been noted that resilience and the “need for redundancy” stands in strong opposition to many ideas about policy-making which equate better designs with efficiency, implying only the minimum possible amount of resources should be allocated to a policy, and which also often emphasize routinization and the replication of standard operating procedures and program elements in order to ensure consistency in program delivery” [33]. As in the literature of policy analysis and new public management, the efficiency of the public service delivery has been widely focused and discussed. However, the public policy needs the consistency and standard operating procedure and there is not much time or space for the robustness of the policy. However, it is necessary to consider some “open space” in the decision-making process. There is, therefore, a need to incorporate a certain extent of slack and redundancy into policy designs and the policy design process, in order to ensure systemic resilience and robustness.

In what is perhaps a further refutation of the NPM approach, health systems that have proven to be relatively more resilient are also ones that have taken a more centralized approach, such as that of South Korea, China, and Singapore. Even in Singapore, policy miss-steps have tended to emerge from the private sector, such as a private laboratory that accidentally disposed of more than 200 COVID-19 test samples [55] or the operators of foreign worker dormitories who overlooked the infection risks that would emerge from these badly-managed dormitories [56,57,58]. In some other contexts of post-disaster resettlement, resilience and livelihood are also used as important measurements in the new normal context. [59]

The COVID-19 pandemic has, therefore, revealed the role of these two characteristics—excess capacity and centralized policy processes—in ensuring the resilience of public service delivery mechanisms during a crisis. Aside from these two characteristics, the existing literature on resilience has also identified other more general attributes of resilience, such as robustness, redundancy, resourcefulness diversity, rapidity, and adaptability [60]. Based on an in-depth literature review of resilience attributes, this paper proposes an analytical framework that incorporates the various resilience attributes and applies this to the study of 16 countries and their pandemic response efforts. We now turn our attention to this framework.

## 3. Research Design and Framework

### 3.1. Analytical Framework

When discussing the perspectives of studying disaster recovery, previous studies conclude economic recovery, social recovery, institutional recovery, and built environment or infrastructure recovery [61,62,63,64,65,66,67]. However, this recovery research framework is not perfectly applicable in the context of COVID-19, as it does not destroy the physical environment such as houses and buildings like other natural disasters but had a profound impact on human society in terms of public health and socio-economic aspects. The economic and social perspectives are the two most important perspectives in studying disaster recovery. Therefore, based on previous disaster resilience studies [68,69,70], this research proposes three perspectives to measure recovery: COVID recovery, economy recovery, and future protection capacity. The first two are used to evaluate the region’s recovery from the devastation caused by the pandemic in the past. We propose the indicator future protection capacity to evaluate the future risk resistance of the region from the destruction of the pandemic in the future as this virus has appeared in multiple variants; the world will still be plagued by the pandemic for a foreseeable long period of time.

**Figure 1:**
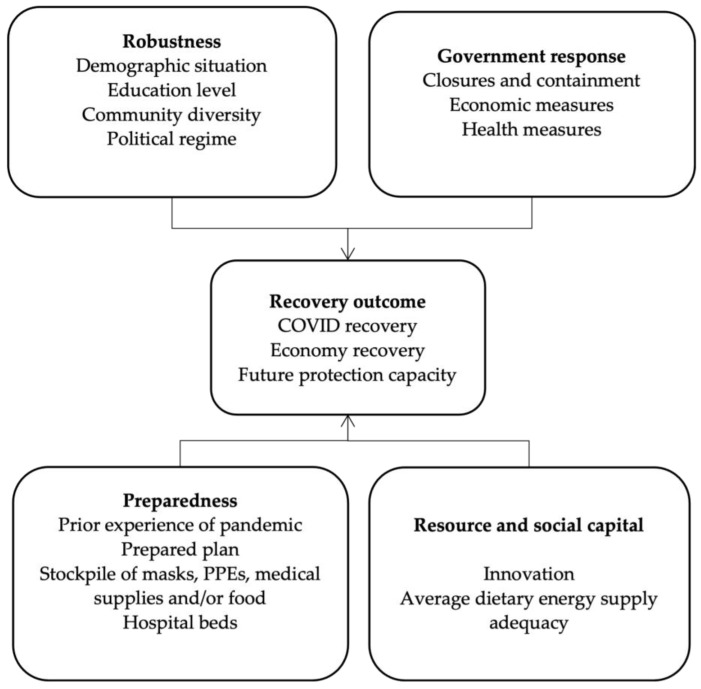
Theoretical framework of resilience attributes and interactions.

For the factors influencing recovery [71,72,73], some fundamental factors are external aids or assistance, disaster damage [74], and social capital functions as the main engines of long-term recovery. Additional factors are pre-disaster and post-disaster planning and socio-economic status, the impacts and disruptions of post-disaster responses and efforts, and macro and micro economic programs and public policies [75,76]. However, the long-term outcomes of external assistance in disaster recovery are still unclear [77]. As widely discussed in the previous literature, resilience evaluation in the context of public policy should be considered the aspects of physical aspect, social aspect, and institutional aspect. Therefore, we choose the different attributes to measure the resilience of public policy through the attributes of robustness, preparedness, resource and social capital, and government response. Based on previous studies, we summarize the four major factors affecting the recovery from COVID-19: robustness, preparedness, resource and social capital, and government response. Robustness is used to measure the ability to address and overcome uncertain disturbances and disasters [59]. As the measurement of robustness, we will normally consider the demographic situation, education level, community diversity, and the political regime of the community. Secondly, preparedness is used in the framework to measure the degree of overcoming the COVID-19 crisis of the communities and the learning capacity of the system. We used physical preparedness and social preparedness to measure (including the storage of PPEs) medical supplies, etc. Thirdly, government response is considered to measure the governmental approach to confront the crisis, which uses the measurements of containment, economic measures, and health measures to measure the capabilities of the government. Last but not least, resourcefulness is one of the important attributes of the resilience framework as widely discussed in the literature [59]. The research attempts to measure the term through innovation and energy supply. The measurement indicators of recovery outcome and its four influencing factors are illustrated in Figure 1.

As Figure 1 shows, our efforts to measure the attribute of “robustness” involved collecting information on cities’ demographic situation, education level, community diversity, and political regime. Robustness is used to measure the flexibility of the system and institutions and it is one of the main attributes of “resilience”. As the robustness of the community is related to the demography, education level, community diversity, and political regime of the community, the paper therefore measures the attributes through the four aspects. In order to measure the indicator “preparedness”, we conclude whether the cities have experienced pandemic before. Additionally, we interviewed local residents pertaining to whether governments had an existing pandemic response plan/procedure before the emergence of COVID-19 and sought to locate the policy document/brief associated with this plan. We also sought to assess whether governments had already built up a stockpile of masks, PPEs, medical supplies, and food. For “resource and social Capital”, we measure it by asking respondents questions pertaining to social innovations and average dietary energy supply adequacy from FAO Food Security Index. Lastly, we assess “government response” by referring to the indicators from the OxCGRT: closures and containment, economic measures, and health measures. The OxCGRT (Oxford COVID-19 Government Response Tracker) database provides a systematic way to track government responses to COVID-19 across countries and sub-national jurisdictions over time. The indices aggregate various measures of government responses to explore whether the government response affects the rate of infection and the recovery of regions.

### 3.2. Fuzzy-Set Qualitative Comparative Analysis (fsQCA) Methodology

Qualitative comparative analysis (QCA) is selected as the research method in this research. Sociologist Charles Ragin first developed it in 1987, inspired by Boolean algebra’s binary logic [78]. QCA mainly helps to examine the causal relationships between several conditions and an outcome of interest. There are three variants of Ragin’s QCA method: crisp-set QCA (csQCA) for dichotomous variables, fuzzy-set QCA (fsQCA) for multichotomous variables, and multi-value QCA (mvQCA) for continuous variables.

fsQCA and csQCA are the most widely used in sociological research. However, the constraint to use only dichotomous variables causes two key problems: information loss and risk of obtaining a large number of contradictory configurations. [79] The more fine-grained information contained in fuzzy-set membership scores provides additional leverage for the selection of the most typical and most deviant cases in the following steps [80]. The selection of the cases used in the fsQCA could be used to measure the combinations of the influencing factors rather than the singular factors to explain the outcomes and results. The selection of the cases could be considered most typical and most deviant accordingly. In this research, the variables are non-binary and have continuous gradations [81]; fsQCA is selected to evaluate the combination of factors to investigate the disaster recovery outcome of COVID-19.

## 4. Research Methodology

### 4.1. Selection of Cases

We select 16 countries worldwide (shown in Table 1) as a sample to analyze the potential factors for recovery from this pandemic. Previous research on community resilience focus on self-organization and resilience of communities playing a decisive role in the post-disaster recovery period [82]. However, in the context of COVID-19, a city’s public service capabilities can have a significant impact on its eventual recovery. To make sure the sample is unbiased, eight cites come from developed countries, and the rest of them come from developing countries. The sample covers almost all of the continents and all of them are important political and economic cities in their regions. Asian cities account for the highest proportion as they have the largest population and most accelerated urbanization area.

### 4.2. Recovery Outcome and Calibrations

As shown in Table 2, this research selects 3 aspects to evaluate a city’s recovery, COVID recovery, economy recovery, and future protection capacity. All the indicators are as of 30 June 2021. If we choose a certain point in 2020 as the cut-off point of our data, due to the different start times of the pandemic and the weak test capacity of different cities, the pandemic data in the sample may include some data deviation, which cannot fully reflect current facts. The reason why we did not use duration period after local onset of pandemic or duration after first wave is that the onset time of the first pandemic is different in each city, and the duration period varies greatly. As a non-public health research, it is difficult for us to accurately define the exact time of the first pandemic in each city. In addition, we did not use the latest date because the virulence of the virus weakens as it mutates, increasing the rate of infection and decreasing the rate of severe illness in all regions. This research tends to focus on the community resilience and recovery during the period when the virus is still virulent, and the rate of severe illness is high.

COVID recovery: This research evaluates the city’s recovery from COVID-19 infection and death, respectively. Our assessment of the recovery situation is to calculate the proportion of the number of infections/deaths in the city on 30 June 2021, to the city’s historical peak data. The smaller the ratio, the better the recovery of the city. Since many countries do not provide city-level pandemic data, we corrected this ratio through the population of the country and the city.

Economy recovery: This research evaluates economy recovery through GDP and unemployment. GDP is the most commonly used indicator to measure the development status of a region. Unemployment is also a measure of how well a place has recovered from a pandemic. Regions that provide enough jobs tend to recover well.

Future protection capacity: At present, coronavirus is still spreading wantonly, and the newly mutated strains are highly contagious, making the duration of the epidemic full of uncertainty. Immunization by vaccination is one of the best ways to avoid large-scale transmission again. Cities with higher vaccination rates can more easily return to normal life and restore economic development.

Raw data collected are calibrated to a number between 0 and 1 according to the requirement of QCA analysis. All calibration follows the formula as shown below. World maximum and minimum are used to calibrate data so that countries not included in the list could also make use of the QCA results obtained from this research.

(1) $$\mathrm{Calibrated}\;\mathrm{data}=\frac{\left(X-W_{\min}\right)}{\left(W_{\max}-W_{\min}\right)}$$

In addition, we must ensure that these four indicators have isotonicity. The closer the calibrated number is to 1, the better the local resilience. Therefore, for some indicators, we subtracted the initial calculated value by 1.

Finally, we take the average of these 3 calibrated indicators as the policy outcome index, as shown in Table 3.

### 4.3. Contributing Factors and Calibrations

As we selected 16 cases as a sample as the fsQCA methods apply to 2^n^ principle, 4 factors can be defined to analyze 16 cases (2^4^ = 16). Given the types of political regimes, economy, external communication, the severity of COVID-19, and the ability of medical care of the cities, we concluded that the most critical four factors of recovery from COVID-19, which are made up of two to four indicators as shown in Table 4.

Robustness: Each city possessed some extent of robustness, and those with specific characteristics are more stable and less likely to be disturbed by the pandemic. People in areas with high life expectancy are in better health, are more immune to the pandemic, and less likely to get infected. High age dependency ratio indicates that the elderly and children, who are vulnerable to infection, account for a large proportion in the city, which deserves government departments’ attention. Government and community policies are easier to implement if the local population has primary education and can read [84]. In diverse communities, there is a big gap in the behavior pattern and way of thinking of residents. Especially in the context of COVID-19, diverse communities are not conducive to the implementation of a unified epidemic prevention policy [85]. Political regime represents the governing ability of a city government, and an efficient government can control the pandemic in a timely manner [86].

Preparedness: If a region has experience dealing with a pandemic and has some material reserves, we think that region can recover quickly from the pandemic. Stockpile of PPEs is used for evaluating the reserves of protective equipment. The medical capacity of a city is an important factor in the government’s consideration of whether to implement lockdown or other policy. If the city has adequate medical resources, it can relax controls appropriately to protect the economy.

Resource and social capital: During the pandemic, the subjective initiative of communities was fully exerted. With limited government resources, many communities organized themselves to deal with the pandemic. It also reflects the resilience of the city. During the raging period of the virus, the production of agricultural products was negatively affected, and the import link was hindered. How the city supplies basic living materials is one of the important evaluation factors to measure the city’s resilience.

Government response: There are three dimensions to measure government response, namely closures and containment, economic measures, and health measures. These three policies can reflect whether the government has taken a response policy after the outbreak and whether the policy is comprehensive.

All of the data are calibrated by Formula 1 and standardized to 0–1, and according to our hypothesis, the closer the index was to 1, the more likely the city was to recover from COVID-19. The averaged indexes of all factors are shown in Table 5.

### 4.4. Research Outcomes

The first step of QCA analysis is to determine the extent to which a combination of factors leads to the outcome; this is known as consistency in fsQCA. According to the QCA method, if the consistency score is 1, it shows that there is a perfect subset relationship between the antecedent condition and outcome, the antecedent condition being considered to be the necessary condition leading to the outcome. However, social science data often do not fully realize the perfect subset relationship, so the standard of 0.95 can be set, which means the variable constitutes a necessary condition for the outcome if the consistency score is greater than 0.95. If the consistency is close to 0.95, the antecedent condition can be considered as an important necessary condition [80].

From Table 6, we can find out that no variable is consistent with 1, so there is no absolutely necessary condition. None of the variables can be considered a necessary condition of recovery, as the consistency of each variable is lower than the critical value of 0.95. It is noteworthy that “X-resources and social capital” have the consistency of 0.94 (very close to 0.95), which indicates that the resource of a city may have a greater influence on recovery, which needs further research and verification. Overall, it is necessary to perform configuration analysis on these condition variables and consider the conjectural synergistic effect of multiple conditions.

According to the principle of QCA, the number of configurations formed by multiple antecedent conditions is logarithmic to the number of selected conditions; that is, for a fuzzy set with k antecedent conditions, 2^k^ configurations can be constructed, and each configuration corresponds to a row in the truth table. This research selects four antecedent conditions, and there will be 16 configurations. Set the consistency threshold to 0.8 (if the result is greater than 0.8, it is 1; if the value is less than 0.8, the result does not exist). In QCA research, 0.8 and 0.75 are the most commonly used thresholds. When the sample size is small, the consistency threshold should be higher, while when the sample size is large, the consistency threshold can be lower. In this research, only 16 samples are selected; thus, we choose 0.8 as the threshold [80]. Meanwhile, the threshold of case frequency is set to 1 (the case result below this value is considered as a logical remainder). According to the above settings, the simplified truth table contains 16 configurations, of which 10 configurations exist in policy outcomes and 6 configurations do not exist as shown in Table 7. There is no contradictory configuration (the configuration with the same condition but the opposite result is called a contradictory configuration). From the truth table, it can be shown that the combination of causes leading to the recovery of cities is diverse, which proves that there is a complex causal relationship between the antecedents and results of recovery from COVID-19 [79].

According to the results of the truth table, fsQCA 3.0 is further used for Boolean minimization. “Standard analyses” identify five distinct patterns comprising different combinations of attributes that determine the resilience of a society and, hence, its ability to recover from the COVID-19 pandemic. These five patterns illustrated in Table 8 together explain the main reasons of good recovery. The first pattern is characterized by a high level of robustness and low level of preparedness. The second pattern features a high level of resources and social capital and low level of preparedness The third pattern refers to cities that possess high levels of robustness and resources and social capital. The fourth pattern features high levels of robustness and government response. The final pattern comprises cities with high levels of resources and social capital, as well as government response. The consistency scores of the five configurations are 0.833491, 0.83596, 0.861196, 0.869426, and 0.83779 respectively.

More specifically, the data reveal that robustness and resources and social capital are essential to disaster recovery in the context of COVID-19. Pattern 1 shows that a city with high robustness could recovery well, even though it may lack sufficient preparedness. In other words, robustness represents resilience to some extent, which makes a city recovery faster and better from a disaster. Compared with other factors, a city’s robustness is an intrinsic characteristic and, hence, not easy to change in the short term.

Another important factor of recovery is resource and social capital, as a lot of social resources need to be consumed at all stages of the pandemic. During a concentrated outbreak of cases, cities need to concentrate medical resources to treat patients and provide residents with sufficient supplies to prevent more infections. After the number of infected people has dropped to a stable level, whether the government has sufficient funds for individuals and enterprises to achieve economic recovery is also a big test. In the future, in order to prevent social order from being overwhelmed by the pandemic again, the governments need to invest funds to support scientific research institutions and pharmaceutical manufacturers to promote vaccines and vaccination. It is shown that cities in pattern 3 and pattern 5 are well-developed in the world or at least in their country.

In addition, we found that low preparedness combines with other contributing factors lead to good recovery in both pattern 1 and 2, while high preparedness does not appear in any of the patterns. Specifically, most case cities in pattern 1 and pattern 2 are from developing countries with a relatively low level of preparedness. The case cities in pattern 3.4.5 are mostly from developed countries, where social capital and other contributing factors have a greater impact on recovery than preparedness. This conclusion suggests that low preparedness is a relatively common phenomenon for cities in developing countries and requires government attention. However, for cities in developed countries, a higher level of preparedness does not contribute resilience as much to other factors such as resources and social capital and government response. Future efforts to improve resilience should focus on raising the level of these conditions

The solution consistency is 0.934025, which means that 93% of cases that meet these five patterns could explain the recovery situation of cities. The solution coverage is 0.786694, which means that the five patterns can explain 86% of the cases. The solution consistency and solution coverage both are higher than the critical value, indicating that the empirical analysis is effective. Perhaps most importantly, our research shows that disaster recovery outcomes as they pertain to COVID-19 do not depend on any single factor. While robustness is critical in influencing COVID-19 disaster recovery outcomes, these often operate in tandem with other resilience attributes or drivers, such as social capital, preparedness, or government response.

## 5. Discussions and Conclusions

Despite the proliferation of research interest in COVID-19 and its policy implications, much of the existing literature on COVID-19 disaster recovery policy has consisted of single-case or small-n studies. There has, as yet, been little effort at developing large-n comparative case studies of countries from multiple regions across the world. This paper has sought to address this gap in the literature by assessing the disaster response efforts and resilience of 16 cities.

By applying fsQCA to these 16 cities, we identify five distinct patterns comprising different combinations factors of robustness, preparedness, resource and social capital, and government response across the cases. As discussed above, the five patterns provide important insights into how different combinations of resilience attributes can give rise to different extents of community resilience and disaster recovery outcomes.

The resilience framework that we have introduced in this paper has served to integrate these resilience attributes in our analysis and evaluation of robustness, preparedness, resource and social capital, and government response in the 16 cases. This has, in turn, allowed us to provide an evidence-based and integrative approach to measuring COVID-19 disaster recovery outcomes across different countries. While our research findings highlight the importance of robustness in facilitating disaster recovery in COVID-19, the resilience attributes of resource and social capital are also crucial to disaster recovery at the city level.

As such, we conclude that community resilience and disaster recovery depend on a combination of factors rather than a single factor. This suggests the need for a broader and more integrative approach to designing policies and institutions for resilience, a la ‘whole-of-government’ [87].

There are some limitations in this research in data selection. We selected daily data and peak data as of 30 June 2021 to present the recovery from COVID-19 of cities. The selection of daily data may not truly reflect infection because of potential statistical and reporting delays. The selection of peak data may overestimate the severity of the pandemic and, thus, the recovery status of the city, which may be caused by untimely reports and accidental deaths caused by urban emergencies.

While we have developed our findings based on the 16 cases, the resilience framework that we introduce to this paper can also be applied to the analysis of other countries and cities from different regional and national contexts [4]. This also points to another important point which we hope to leave the reader with, i.e., the need for further research on the nature and driver of resilience in the face of COVID-19. At this time of writing, the world remains far from a full recovery from the COVID-19 pandemic, and it is difficult to predict its future trend [88]. Rather, global recovery remains patchy, with more resilient and high-performing countries such as Israel and Singapore exhibiting strong economic recovery and vaccination rates while other such as Malaysia and India continue to grapple with high infection rates and inadequate access to vaccines.

In light of these challenges, policymakers and governments across the world bear the heavy burden of designing effective policies to improve resilience in the face of future pandemics [89,90]. This will require more robust policy designs and institutions, as well as facilitating social networks and ground-up initiatives through procedural policy design [33,91]. It is on this note that we hope our paper can prompt further research and discourse on the nature of community resilience, particularly as it pertains to COVID-19 and across different policy systems and countries across the world.

## Figures and Tables

**Table 1:** List of case cities.

No	ISO	Continent	Type	Country	City
1	ZWE	Africa	Developing	Zimbabwe	Harare
2	USA	North America	Developed	United States	Cincinnati, Ohio
3	FRA	Europe	Developed	France	Paris
4	KHM	Asia	Developing	Cambodia	Phnom Penh
5	MWI	Africa	Developing	Malawi	Lilongwe
6	MMR	Asia	Developing	Myanmar	Mandalay
7	GBR	Europe	Developed	United Kingdom	London
8	NZL	Oceania	Developed	New Zealand	Auckland
9	RUS	Europe	Developed	Russian Federation	Moscow
10	TZA	Africa	Developing	Tanzania	Dar Es Salaam
11	IND	Asia	Developing	India	Delhi
12	JPN	Asia	Developed	Japan	Tokyo
13	KOR	Asia	Developed	Korea, Rep.	Seoul
14	SGP	Asia	Developed	Singapore	Singapore
15	CHN	Asia	Developing	China	Hangzhou
16	PAK	Asia	Developing	Pakistan	Islamabad

**Table 2:** Recovery outcome.

Recovery Outcome	Indicators	Explanation	Source
COVID recovery	Infection recovery	The proportion of the number of infections in the city on 30 June 2021, to the city’s historical peak data.	Ministry of Health of each country or city, JHU, and other databases
	Death recovery	The proportion of the number of deaths in the city on 30 June 2021, to the city’s historical peak data.	
Economy recovery	GDP recovery	GDP growth in 2021 compared to 2019	IMF (2021, predicted); World Bank (2020)
	Unemployment recovery	The unemployment rate growth in 2020 compared to 2019.	World Bank
Future protection capacity	Vaccinations per hundred	This research applies the national data to the city level.	OWID database

**Table 3:** Summary of indicators included in recovery outcome index.

City	COVID Recovery		Economy Recovery		Future Protection Capacity
	Infection Recovery	Death Recovery	GDP Growth	Unemployment Rate	Vaccine Coverage
Harare	0.00	0.05	0.62	0.88	0.06
Cincinnati	0.98	1.00	0.69	0.00	0.65
Paris	1.00	0.98	0.51	1.00	0.53
Phnom Penh	0.00	0.00	0.54	1.00	0.28
Lilongwe	0.00	0.56	0.71	0.94	0.01
Mandalay	0.00	0.23	0.00	0.75	0.04
London	0.69	0.98	0.45	0.91	0.74
Auckland	0.97	1.00	0.71	0.93	0.15
Moscow	1.00	0.00	0.65	0.79	0.18
Dar es Salaam	1.00	1.00	0.94	1.00	0.00
Delhi	0.99	0.96	0.65	0.63	0.15
Tokyo	0.69	0.99	0.50	0.91	0.26
Seoul	0.00	0.87	0.72	0.97	0.25
Singapore	0.99	1.00	0.60	0.57	0.39
Hangzhou	1.00	1.00	1.00	0.95	0.56
Islamabad	0.17	0.00	0.72	0.89	0.04

Notes: The higher the number, the better the recovery in this perspective. Because we used global data to calibrate our calculations, there are many 0/1 values, indicating that these cities are recovering poorly/well on a global scale.

**Table 4:** Contributing factors.

Contributing Factors	Indicators	Explanation	Source
Robustness	demographic situation	Demographic situation contains two aspects, life expectancy and age dependency ratio. The age dependency ratio means that the non-working population is divided by the working population. National data are used to represent the city level.	World Bank
	education level	School enrollment (primary) is used to measure the education level of the city. National data are used to represent the city level.	World Bank
	community diversity	We use the community diversity index, which consists of ethnicity, languages, and the size of the migrant community, to measure the city’s diversity.	Questionnaire
	political regime	The research uses two indicators to measure the political situations of the cities. One is the polity score, and the other is Corruption Perception Index. These two indicators are also for the national level, and we believe it is reasonable to apply them to the cities.	Center for Systemic Peace 2017 (polity score); World Bank (Corruption Perception Index)
Preparedness	prior experience of pandemic	This indicator measures whether the country has experience in dealing with pandemics such as SARS and Ebola in the past. National data are applied to the city level.	Ma, Rogers, and Zhou (2020) [83]
	prepared plan	We searched public government data, asked local residents for information, and gave each location a score on a five-point scale, with higher scores indicating the better reserves.	Questionnaire
	stockpile of masks, PPEs, medical supplies, and/or food		Questionnaire
	hospital beds per 1000 people	National-level data applied to the city level.	WHO Global Health Observatory
Resources and social capital	innovation	This indicator is measured by five grades from 1 to 5 in city level.	Questionnaire
	average dietary energy supply adequacy	This research uses the data of 2018–2020 3-year average to reflect the basic food supply capacity of the city.	FAO Food Security Index
Government response	closures and containment	Seven indicators are used to measure the policy of closures and containment of a city. They are school closing, workplace closing, cancel public events, restrictions on gatherings, close public transport, stay-at-home requirements, restrictions on internal movement, international travel controls.	OxCGRT database
	economic measures	Three indicators are selected to measure economic policy implication. Income support records if the government is covering the salaries or providing direct cash payments, universal basic income, or similar, of people who lose their jobs or cannot work. Debt/contract relief judges if government is freezing financial obligations. Fiscal measures figure out what economic stimulus policies are adopted.	
	health measures	Testing policy finds out who can get tested. Contact tracing records whether governments are doing contact tracing. Emergency investment in health care measures short-term spending. Investment in vaccines announces public spending on vaccine development. Vaccination policy records policies for vaccine delivery for different groups. Protection of elderly people records policies for protecting elderly people in long term care facilities and/or the community and home setting.	

**Table 5:** Averaged indexes of contributing factors.

City	Y-Recovery Outcome	X-Robustness	X-Preparedness	X-Resource and Social Capital	X-Government Response
Harare	0.2780	0.5224	0.3759	0.3914	0.4745
Cincinnati, Ohio	0.6609	0.5634	0.5990	0.7648	0.7722
Paris	0.7567	0.4839	0.6090	0.6336	0.6453
Phnom Penh	0.3498	0.5232	0.5101	0.8516	0.5818
Lilongwe	0.3747	0.5438	0.3680	0.6516	0.4218
Mandalay	0.1776	0.5333	0.5128	0.4633	0.6174
London	0.7514	0.5852	0.7409	0.9023	0.7286
Auckland	0.6501	0.5351	0.5930	0.6867	0.4966
Moscow	0.4659	0.6332	0.3329	0.5219	0.6166
Dar es Salaam	0.6563	0.3846	0.4061	0.5164	0.2606
Delhi	0.5898	0.5175	0.4528	0.3203	0.6736
Tokyo	0.6036	0.6158	0.6486	0.7242	0.7224
Seoul	0.5088	0.7424	0.7878	0.8336	0.5444
Singapore	0.6573	0.5670	0.6415	0.9102	0.8246
Hangzhou	0.8451	0.5273	0.6274	0.8180	0.7485
Islamabad	0.3096	0.4001	0.4547	0.6164	0.6432

**Table 6:** Necessary condition analysis.

Contributing Factors	Consistency	Coverage
X-robustness	0.84	0.83
~X-robustness	0.73	0.87
X-preparedness	0.88	0.88
~X-preparedness	0.68	0.80
X-resources and social capital	0.94	0.76
~X-resources and social capital	0.52	0.84
X-government response	0.91	0.80
~X-government response	0.62	0.86

Notes: According to the QCA methodology, “~” indicates that the condition variable is encoded as 0.

**Table 7:** Truth table analysis.

X-Robustness	X-Preparedness	X-Resources and Social Capital	X-Government Response	Number of Cases	Raw Consist.
1	1	1	1	7	0.908005
1	0	0	0	1	0.891111
0	0	1	0	1	0.904079
1	0	1	0	1	0.887203
1	1	1	0	1	0.923312
1	0	0	1	1	0.87725
1	1	0	1	1	0.88835
0	0	1	1	1	0.865182
1	0	1	1	1	0.878325
0	1	1	1	1	0.898076

Notes: All configurations of the contributing variables and result variables are presented in the form of a table, that is, the truth table. The rows of the truth table represent various logical combinations of contributing conditions and the results that result from the different combinations.

**Table 8:** fsQCA outcomes of the combination of factors.

Combination of Contributing Factors	Raw Coverage	Unique Coverage	Consistency	Cities
X-robustness*~X-preparedness	0.6497	0	0.833491	Moscow (RUS) Lilongwe (MWI) Harare (ZWE) Delhi (IND)
~X-preparedness*X-resources and social capital	0.642135	0.0152655	0.835962	Lilongwe (MWI) Islamabad (PAK) Moscow (RUS) Dar es Salaam (TZA)
X-robustness*X-resources and social capital	0.814622	0.00445706	0.861196	Seoul (KOR) Tokyo (JPN) London (GBR) Singapore (SGP) Cincinnati, Ohio (USA) Lilongwe (MWI) Auckland (NZL) Hangzhou (CHN) Phnom Penh (KHM) Moscow (RUS)
X-robustness*X-government response	0.818636	0	0.869426	Moscow (RUS) Tokyo (JPN) London (GBR) Singapore (SGP) Cincinnati, Ohio (USA) Seoul (KOR) Mandalay (MMR) Hangzhou (CHN) Phnom Penh (KHM) Delhi (IND)
X-resources and social capital*X-government response	0.877113	0.0813074	0.83779	Singapore (SGP) Cincinnati, Ohio (USA) Hangzhou (CHN) London (GBR) Tokyo (JPN) Paris (FRA) Islamabad (PAK) Phnom Penh (KHM) Seoul (KOR) Moscow (RUS)
solution coverage: 0.934025				
solution consistency: 0.786694				

Notes: The symbol “*” represents the intersection relation of “and”; The symbol “~” represents the logical relation of “not”, that is, the code of the condition variable is 0; “Raw Coverage” represents the proportion of cases that can be explained by this configuration. Since the same result can be reflected by multiple configuration patterns, raw coverage is the main index of analysis, showing the adequacy. “Unique Coverage” represents the percentage of cases that can only be explained by this configuration. “Solution Coverage” represents the percentage of cases that can be explained by the solution as a whole.

## Data Availability

Data are contained within the article.
